# Intersphincteric resection following robotic-assisted versus laparoscopy-assisted total mesorectal excision for middle and low rectal cancer: a multicentre propensity score analysis of 1571 patients

**DOI:** 10.1097/JS9.0000000000001053

**Published:** 2024-01-19

**Authors:** Yuchen Guo, Liang He, Weidong Tong, Shuangyi Ren, Zhaocheng Chi, Ke Tan, Bo Wang, Chunxiao Lie, Quan Wang

**Affiliations:** a Department of Gastrocolorectal Surgery, General Surgery Center, The First Hospital of Jilin University; b Jilin Provincial Tumour Hospital, Changchun; c Daping Hospital and the Research Institute of Surgery of the Third Military Medical University, Chongqing Municipality; d Second Affiliated Hospital of Dalian Medical University, Dalian, China

**Keywords:** intersphincteric resection, laparoscopy, rectal cancer, robot, total mesorectal excision

## Abstract

**Background:**

Robotic-assisted total mesorectal excision (RaTME) may be associated with reduced conversion to an open approach and a higher rate of complete total mesorectal excision (TME); however, studies on its advantages in intersphincteric resection (ISR) are inadequate.

**Materials and methods:**

This retrospective multicenter cohort study enroled consecutive patients who underwent RaTME and laparoscopy-assisted total mesorectal excision (LaTME) at four medical centres between January 2020 and March 2023. Propensity score matching (PSM), inverse probability of treatment weight (IPTW), and multivariate logistic regression analyses were performed. The primary outcome was the ISR rate. Secondary outcomes were coloanal anastomosis (CAA), conversion to open surgery, conversion to transanal TME, abdominoperineal resection, postoperative morbidity and mortality within 30 days, and pathological outcomes.

**Results:**

Among the 1571 patients, 1211 and 450 underwent LaTME and RaTME, respectively, with corresponding ISR incidences of 5.3% and 8.4% (*P*=0.024). After PSM and IPTW, RaTME remained associated with higher ISR rates (4.5% versus 9.4%, *P*=0.022 after PSM; 4.9% versus 9.2, *P*=0.005 after IPTW). This association remained in multivariate analysis after adjusting for other confounding factors. RaTME was further associated with a higher CAA rate, longer operating time, and higher hospitalization expenses.

**Conclusions:**

RaTME may facilitate ISR in middle and low rectal cancers, showing an independent association with a higher ISR incidence, with pathological outcomes and complications comparable to those of LaTME. However, it may also require a longer operating time and incur higher hospitalization expenses.

## Introduction

Highlights

- Compared with laparoscopy-assisted total mesorectal excision (LaTME), robotic-assisted total mesorectal excision (RaTME) was independently associated with a higher rate of intersphincteric resection and coloanal anastomosis.
- The present study indicated that RaTME was superior to LaTME in ultra-low anterior resection for low rectal cancer.
- RaTME was comparable to LaTME in pathological outcomes and 30-day postoperative complications.

Total mesorectal excision (TME) for middle and lower rectal cancer remains a challenging procedure, with surgeons commonly encountering limited vision and operating space during low anterior resection. These problems may result in an incomplete TME^1,2^, unexpected surgical injuries, or the need for conversion to laparotomy. In extreme cases, these problems may lead to conversion to abdominoperineal resection (APR) and a reduction in the anus-preservation rate. Since its initial application in proctectomy, the advantages of the application of robotic techniques in TME have been extensively investigated. Robotic-assisted total mesorectal excision (RaTME) has several advantages, and studies focusing on the safety and efficacy of the robotic technique have previously evaluated outcomes such as completion of the TME, circumferential resection margin (CRM) positivity, and conversion rate^3–7^. Several retrospective studies have indicated that RaTME can significantly reduce the need for a transanal resection approach, which may reflect its technical advantages for lower rectal cancer^8,9^. However, few studies have discussed its advantages in the field of intersphincteric resection (ISR) and anus-preserving surgery, which may also be improved by robotic surgery, as its three-dimensional vision and flexible robotic arms have been indicated to improve surgical quality and facilitate pelvic resection in proctectomy^10,11^. Herein, we aimed to determine the efficacy of RaTME in achieving ISR and preserving the anus compared to that of laparoscopy-assisted total mesorectal excision (LaTME) in middle and low rectal cancers.

## Materials and methods

### Study design and ethics

This was a multicenter, retrospective, propensity score-based cohort study that enroled 1571 patients from four medical centres in China. This study was approved by the Ethics Committee of our medical centre (approval number: 23K163-001). The detailed procedures were explained to all patients, and written informed consent was obtained prior to treatment. This work has been reported in line with the STROCSS criteria^12^. The research was retrospectively registered in ResearchRegistry.com. The Unique Identifying Number is researchregistry9626.

### Population

We retrospectively analyzed patients diagnosed with rectal cancer who underwent LaTME or RaTME in four centres (First Hospital of Jilin University in Jilin province, Jilin Provincial Tumor Hospital in Jilin province, Daping Hospital and the Research Institute of Surgery of the Third Military Medical University in Chongqing Municipality, the Second Affiliated Hospital of Dalian Medical University in Liaoning province) between January 2020 and March 2023.

The inclusion criteria were: (1) aged 18–90 years; (2) diagnosed with rectal cancer by pathological biopsy; (3) tumour located at a height of less than 10 cm from the anal verge but above the intersphincteric groove. When the distances from the anal verge to the tumour measured using the different methods (evaluated by preoperative rectal magnetic resonance imaging, endorectal ultrasound, colonoscopy, or digital rectal examination) were different, the minimum distance was used. (4) Treatment with radical LaTME or RaTME.

The exclusion criteria were: (1) multiple primary cancers; (2) abdominal contrast-enhanced and chest computed tomography (CT) (or positron emission tomography-CT) revealing distal metastasis or distal metastasis discovered during surgical exploration or confirmed by postoperative pathologic examination; (3) suspected violation of the levator ani muscle and external anal sphincter before surgery (evaluated by rectal magnetic resonance imaging); (4) patients with complete bowel obstruction, digestive tract haemorrhage, digestive tract perforation, or any other tumour-associated complications that required an emergency operation; (5) combined organ resection during the surgery; and (6) pathological stage T4 in multicenter database.

### Exposure

All the patients underwent radical resection for rectal cancer. Five surgeons participated in this study. All surgeons used both LaTME and RaTME. All surgeons were specialized experts in colorectal surgery and had performed at least 40 cases of RaTME before enrolment. Surgeons communicated with the patients about the details of both LaTME and RaTME before the operation and revealed the advantages and disadvantages of each surgical technique. Patients decided which surgical technique they want to receive.

In the LaTME group, low anterior resection and mesorectal excision procedures were completed laparoscopically. In the RaTME group, low anterior resection and TME were completed with the assistance of a robot (da Vinci Xi Surgical System). There were no limitations on the ligation site of the inferior mesenteric artery (high or low tie), D3 or D2 dissection, splenic flexure mobilization, preventive stoma, or the use of transabdominal or transanal drainage tubes. All of the above procedures were permissible and could be performed at the discretion of the surgeon.

APR was performed in both the LaTME and RaTME groups when surgeons considered it necessary to achieve R0 resection. Conversion to TaTME was considered for LaTME or RaTME if the surgeons experienced difficulty in completing the TME via the transabdominal approach. Conversion to open surgery was considered for LaTME or RaTME if the surgeons experienced difficulty in performing radical resection or in dealing with adverse events during surgery.

### Outcomes and definitions

The primary outcome was the incidence of ISR. The ISR was defined according to the definition provided by a Japanese study group^13^. ISR was determined in cases in which the distal resection margin was from the dentate line to the intersphincteric groove. Secondary outcomes included coloanal anastomosis (CAA), conversion to open surgery, conversion to TaTME, incidence of APR, postoperative morbidity and mortality within 30 days after surgery, and pathological outcomes.

CAA was defined as an anastomosis of the distal colon and surgical anal canal; in CAA, the distal resection margin is located below the hiatus of the levator ani muscle. Conversion to open surgery was defined as an abdominal incision larger than necessary for specimen extraction. Conversion to TaTME was defined as a TME that could not be completed via the transabdominal approach and had to be instead completed by a transanal approach with the help of an imaging system and endoscopic instruments. It should be noted that if the TME was completely performed via the transabdominal approach, simply dissecting the internal sphincter and intersphincteric space or finishing the coloanal anastomosis via the transanal approach was not classified as TaTME. The 30-day postoperative morbidity and mortality were evaluated according to the Clavien–Dindo classification^14^. The pathological outcomes included CRM positivity rate, distal resection margin positivity rate, tumour volume, number of lymph nodes, and pathological TNM stage. The detailed definitions of these variables are provided in the Supplemental Material, Supplemental Digital Content 1, http://links.lww.com/JS9/B740.

### Statistical analysis

Categorical variables are presented as numbers with percentages and were analyzed using the χ^2^ test, Fisher’s exact test, or logistic regression. Continuous data with normal distribution are presented as the mean (standard deviation) and were analyzed using Student’s *t*-test. Continuous variables with non-normal distributions were reported as medians with interquartile ranges and were compared using the Mann–Whitney U test.

Propensity score matching (PSM) and inverse probability of treatment weight (IPTW) were used to balance differences in baseline characteristics owing to possible selection bias among patients who underwent LaTME vs. RaTME. The variables included in the propensity model were sex, age, preoperative concurrent chemoradiotherapy, distance from the anal verge, ASA, BMI, pathological stage T, pathological stage N, and medical centre. LaTME was 1:1 matched to RaTME according to the nearest neighbour method, with a caliper width of 0.02. For the IPTW method, a pseudo-population was created by weighting the inverse probability of a patient undergoing RaTME versus LaTME based on the propensity score^15^.

The risk factors for ISR were analyzed using multivariate logistic regression. We further conducted a subgroup analysis of the entire cohort. Univariate and multivariate logistic regression analyses were used in the subgroup analysis. Medical centre, sex, age, ASA, application of neoadjuvant radiochemotherapy, height of tumour from the anal verge, BMI, conversion to TaTME, pathological stage T, and pathological stage N were factors adjusted for in the multivariable analysis. The effect size was estimated using odds ratios (ORs) with 95% CIs. Sensitivity analysis was performed using a multivariate analysis to adjust for confounding factors in the unmatched, PSM, and IPTW cohorts.

In the statistical analyses, multiple imputations were used to complete missing values. All statistical analyses were performed using IBM SPSS Statistics for Mac, version 26 (IBM Corp.) and R for Mac, version 4.1.2 (R Foundation for Statistical Computing). Significance was set at a two-sided *P* value of 0.05.

## Results

### Patient and treatment characteristics

In total, 2164 patients were identified as potential candidates, of whom 593 were excluded; the reasons for exclusion are shown in Figure S1. The remaining 1571 patients were included in the analysis, of whom 408 were from the First Hospital of Jilin University, 57 from Jilin Provincial Tumor Hospital, 852 from Daping Hospital and the Research Institute of Surgery of the Third Military Medical University, and 254 from the Second Affiliated Hospital of Dalian Medical University. Of the 1571 patients, 1121 underwent LaTME and 450 underwent RaTME. After PSM, 662 patients were enroled for the analysis of primary and secondary outcomes, with 331 patients included in each matched group. In the IPTW cohort, 1569.8 cases were in the LaTME group, and 1574 cases were in the RaTME group. The characteristics of the patients in the entire cohort, PSM cohort, and IPTW cohort are shown in Table 1. The population and perioperative treatment of each medical centre after PSM are listed in Table S1

**Table 1:** Patients’ clinical characteristics before and after matching.

	Entire cohort				PSM cohort				IPTW cohort		
Variables	Total (*n* = 1571)	LaTME (*n* = 1121)	RaTME (*n* = 450)	*P*	Total (*n*=662)	LaTME (*n*=331)	RaTME (*n*=331)	*P*	LaTME (*n*= 1569.8)	RaTME (*n*=1574.0)	*P*
Sex, *n* (%)											
Male	946 (60.2)	664 (59.2)	282 (62.7)	0.23	410 (61.9)	203 (61.3)	207 (62.5)	0.81	943.7 (60.1)	982.8 (62.4)	0.495
Female	625 (39.8)	457 (40.8)	168 (37.3)		252 (38.1)	128 (38.7)	124 (37.5)		626.1 (39.9)	591.2 (37.6)	
Age, *n* (%)											
≤65 years	945 (60.2)	670 (59.8)	275 (61.1)	0.664	410 (61.9)	207 (62.5)	203 (61.3)	0.81	940.2 (59.9)	1001.8 (63.6)	0.267
>65 years	626 (39.8)	451 (40.2)	175 (38.9)		252 (38.1)	124 (37.5)	128 (38.7)		629.6 (40.1)	572.2 (36.4)	
Albumin, *n* (%)											
>30 g/l	1548 (98.5)	1104 (98.5)	444 (98.7)	0.967	654 (98.8)	328 (99.1)	326 (98.5)	0.725^a^	1548.8 (98.7)	1559.6 (99.1)	0.475
≤30 g/l	23 (1.5)	17 (1.5)	6 (1.3)		8 (1.2)	3 (0.9)	5 (1.5)		21.0 (1.3)	14.4 (0.9)	
DAVT, *n* (%)											
>5 cm	836 (53.2)	567 (50.6)	269 (59.8)	0.001	391 (59.1)	196 (59.2)	195 (58.9)	>0.999	830.0 (52.9)	870.1 (55.3)	0.493
≤5 cm	735 (46.8)	554 (49.4)	181 (40.2)		271 (40.9)	135 (40.8)	136 (41.1)		739.8 (47.1)	703.9 (44.7)	
BMI, *n* (%)											
<24 kg/m^2^	791 (50.4)	595 (53.1)	196 (43.6)	<0.001	302 (45.6)	151 (45.6)	151 (45.6)	>0.999	791.1 (50.4)	770.7 (49.0)	0.682
≥24 kg/m^2^	780 (49.6)	526 (46.9)	254 (56.4)		360 (54.4)	180 (54.4)	180 (54.4)		778.7 (49.6)	803.3 (51.0)	
CCRT, *n* (%)											
No	1385 (88.2)	981 (87.5)	404 (89.8)	0.242	586 (88.5)	292 (88.2)	294 (88.8)	0.903	1380.6 (87.9)	1389.0 (88.2)	0.888
Yes	186 (11.8)	140 (12.5)	46 (10.2)		76 (11.5)	39 (11.8)	37 (11.2)		189.2 (12.1)	185.0 (11.8)	
ASA, *n* (%)											
I–II	1398 (89)	1009 (90)	389 (86.4)	0.051	578 (87.3)	290 (87.6)	288 (87)	0.907	1400.3 (89.2)	1426.8 (90.6)	0.405
III–IV	173 (11)	112 (10)	61 (13.6)		84 (12.7)	41 (12.4)	43 (13)		169.5 (10.8)	147.2 ( 9.4)	
Pathological stage T, *n* (%)											
0	36 (2.3)	26 (2.3)	10 (2.2)	0.712	15 (2.3)	9 (2.7)	6 (1.8)	0.655^a^	33.9 (2.2)	32.3 (2.1)	0.999
1	126 (8)	88 (7.9)	38 (8.4)		52 (7.9)	29 (8.8)	23 (6.9)		125.3 ( 8.0)	118.7 ( 7.5)	
2	425 (27.1)	294 (26.2)	131 (29.1)		168 (25.4)	79 (23.9)	89 (26.9)		420.9 (26.8)	422.4 (26.8)	
3	965 (61.4)	698 (62.3)	267 (59.3)		421 (63.6)	212 (64)	209 (63.1)		970.5 (61.8)	982.2 (62.4)	
Tis	19 (1.2)	15 (1.3)	4 (0.9)		6 (0.9)	2 (0.6)	4 (1.2)		19.2 (1.2)	18.4 (1.2)	
Pathological stage N, *n* (%)											
0	983 (62.6)	701 (62.5)	282 (62.7)	0.035	413 (62.4)	208 (62.8)	205 (61.9)	0.776	983.7 (62.7)	987.7 (62.7)	0.983
1	413 (26.3)	308 (27.5)	105 (23.3)		161 (24.3)	77 (23.3)	84 (25.4)		410.5 (26.2)	405.2 (25.7)	
2	175 (11.1)	112 (10)	63 (14)		88 (13.3)	46 (13.9)	42 (12.7)		175.5 (11.2)	181.2 (11.5)	
Pathological stage TNM, *n* (%)											
0	19 (1.2)	15 (1.3)	4 (0.9)	0.93	6 (0.9)	2 (0.6)	4 (1.2)	0.663^a^	19.2 (1.2)	18.4 (1.2)	0.898
I	446 (28.4)	315 (28.1)	131 (29.1)		178 (26.9)	93 (28.1)	85 (25.7)		459.2 (29.3)	424.3 (27.0)	
II	486 (30.9)	347 (31)	139 (30.9)		215 (32.5)	104 (31.4)	111 (33.5)		474.0 (30.2)	518.6 (32.9)	
III	588 (37.4)	420 (37.5)	168 (37.3)		249 (37.6)	123 (37.2)	126 (38.1)		586.1 (37.3)	586.3 (37.3)	
pCR	32 (2)	24 (2.1)	8 (1.8)		14 (2.1)	9 (2.7)	5 (1.5)		31.3 (2.0)	26.4 (1.7)	
Medical centre, *n* (%)											
First Hospital of Jilin University	408 (26)	268 (23.9)	140 (31.1)	< 0.001	260 (39.3)	128 (38.7)	132 (39.9)	0.991	407.5 (26.0)	404.5 (25.7)	0.999
Jilin Provincial Tumour Hospital	57 (3.6)	27 (2.4)	30 (6.7)		38 (5.7)	19 (5.7)	19 (5.7)		55.7 (3.5)	57.3 (3.6)	
Daping Hospital and the Research Institute of Surgery of the Third Military Medical University	852 (54.2)	747 (66.6)	105 (23.3)		213 (32.2)	108 (32.6)	105 (31.7)		852.4 (54.3)	858.4 (54.5)	
Second Affiliated Hospital of Dalian Medical University	254 (16.2)	79 (7)	175 (38.9)		151 (22.8)	76 (23)	75 (22.7)		254.2 (16.2)	253.9 (16.1)	

ASA, American Society of Anesthesiologists; CCRT, concurrent chemoradiotherapy;. DAVT, distance from the anal verge to the tumour; IPTW, inverse probability of treatment weight; LaTME, laparoscopy-assisted total mesorectal excision; pCR, pathological complete regression; PSM, propensity score matching RaTME, robotic-assisted total mesorectal excision.

a Fisher exact test.

### Differences in ISR rate between LaTME and RaTME

Before PSM, 97 (6.2%) patients underwent ISR, including 59 in the LaTME group and 38 in the RaTME group (5.3% versus 8.4%; *P*=0.024). In the study following PSM, 46 patients (6.9%) underwent ISR. It comprised 15 patients (4.5%) from the LaTME group and 31 patients (9.4%) from the RaTME group, revealing a statistically significant difference (*P*=0.022). Analysis after IPTW indicated similar results, with an ISR rate of 4.9% in the LaTME group and 9.2% in the RaTME group (*P*=0.005) (Table 2). In the sensitivity analysis, we performed multivariate analysis to adjust for the confounding factors. RaTME was associated with higher ISR after adjustment in the entire cohort [OR, 4.76 (95% CI, 2.46–9.21), *P*<0.001], PSM cohort [OR, 3.69 (95% CI, 1.63–8.35), *P*=0.002], and IPTW cohort [OR, 4.25 (95% CI, 2.91–6.23), *P*<0.001] (Table 3).

**Table 2:** Comparison of primary outcomes and secondary outcomes between LaTME and RaTME.

	Entire cohort				PSM cohort				IPTW cohort		
Variables	Total (*n*=1571)	LaTME (*n*=1121)	RaTME (*n*=450)	*P*	Total (*n*=662)	LaTME (*n*=331)	RaTME (*n*=331)	*P*	LaTME (*n*= 1569.8)	RaTME (*n*=1574.0)	*P*
ISR, *n* (%)											
No	1474 (93.8)	1062 (94.7)	412 (91.6)	0.024	616 (93.1)	316 (95.5)	300 (90.6)	0.022	1493.2 (95.1)	1428.7 (90.8)	0.005
Yes	97 (6.2)	59 (5.3)	38 (8.4)		46 (6.9)	15 (4.5)	31 (9.4)		76.6 (4.9)	145.3 (9.2)	
CAA, *n* (%)											
No	1448 (92.2)	1046 (93.3)	402 (89.3)	0.011	601 (90.8)	311 (94)	290 (87.6)	0.007	1472.7 (93.8)	1400.8 (89.0)	0.004
Yes	123 (7.8)	75 (6.7)	48 (10.7)		61 (9.2)	20 (6)	41 (12.4)		97.1 (6.2)	173.2 (11.0)	
APR, *n* (%)											
No	1346 (85.7)	953 (85)	393 (87.3)	0.268	582 (87.9)	293 (88.5)	289 (87.3)	0.721	1341.7 (85.5)	1295.1 (82.3)	0.252
Yes	225 (14.3)	168 (15)	57 (12.7)		80 (12.1)	38 (11.5)	42 (12.7)		228.0 (14.5)	278.9 (17.7)	
Anastomotic method, *n* (%)											
Stapler	1208 (76.9)	856 (76.4)	352 (78.2)	<0.001	524 (79.2)	267 (80.7)	257 (77.6)	0.032	1211.8 (77.2)	1145.7 (72.8)	0.039
Handsewn	100 (6.4)	59 (5.3)	41 (9.1)		47 (7.1)	15 (4.5)	32 (9.7)		76.6 (4.9)	149.5 (9.5)	
No anastomosis	263 (16.7)	206 (18.4)	57 (12.7)		91 (13.7)	49 (14.8)	42 (12.7)		281.4 (17.9)	278.8 (17.7)	
Preventive stoma, *n* (%)											
No	652 (41.5)	435 (38.8)	217 (48.2)	<0.001	336 (50.8)	186 (56.2)	150 (45.3)	0.007	689.5 (43.9)	671.2 (42.6)	0.711
Yes	919 (58.5)	686 (61.2)	233 (51.8)		326 (49.2)	145 (43.8)	181 (54.7)		880.3 (56.1)	902.8 (57.4)	
Conversion to TaTME, *n* (%)											
No	1410 (89.8)	982 (87.6)	428 (95.1)	<0.001	609 (92)	299 (90.3)	310 (93.7)	0.152	1399.8 (89.2)	1463.8 (93.0)	0.074
Yes	161 (10.2)	139 (12.4)	22 (4.9)		53 (8)	32 (9.7)	21 (6.3)		170.0 (10.8)	110.2 (7.0)	
Conversion to open, *n* (%)											
No	1546 (98.4)	1098 (97.9)	448 (99.6)	0.038	653 (98.6)	324 (97.9)	329 (99.4)	0.177^a^	1541.7 (98.2)	1562.0 (99.2)	0.249
Yes	25 (1.6)	23 (2.1)	2 (0.4)		9 (1.4)	7 (2.1)	2 (0.6)		28.1 (1.8)	12.0 (0.8)	
Operating time, min^b^											
	165 (135,201)	162 (130,200)	172 (147.25,205)	<0.001	165.5 (135.25, 204)	160 (125, 200.5)	172 (146.5, 205)	< 0.001	168.86 (54.26)	187.49 (53.49)	<0.001
Intraoperative bleeding, *n* (%)											
No	1531 (97.5)	1088 (97.1)	443 (98.4)	0.161	647 (97.7)	323 (97.6)	324 (97.9)	>0.999	1531.7 (97.6)	1517.9 (96.4)	0.36
Yes	40 (2.5)	33 (2.9)	7 (1.6)		15 (2.3)	8 (2.4)	7 (2.1)		38.1 (2.4)	56.1 (3.6)	
Overall complications, *n* (%)											
No	1186 (75.5)	834 (74.4)	352 (78.2)	0.126	523 (79)	268 (81)	255 (77)	0.252	1203.6 (76.7)	1149.7 (73.0)	0.232
Yes	385 (24.5)	287 (25.6)	98 (21.8)		139 (21)	63 (19)	76 (23)		366.2 (23.3)	424.3 (27.0)	
Minor complications, *n* (%)											
I–II	279 (17.8)	209 (18.6)	70 (15.6)	0.854	100 (71.9)	44 (69.8)	56 (73.7)	0.755	263.8 (72.0)	300.8 (70.9)	0.859
Major complications, *n* (%)											
≥III	105 (27.3)	77 (26.9)	28 (28.6)		39 (28.1)	19 (30.2)	20 (26.3)		102.4 (28.0)	123.5 (29.1)	
Reoperation, *n* (%)											
No	1540 (98)	1102 (98.3)	438 (97.3)	0.293	651 (98.3)	327 (98.8)	324 (97.9)	0.543	1545.6 (98.5)	1527.0 (97.0)	0.122
Yes	31 (2)	19 (1.7)	12 (2.7)		11 (1.7)	4 (1.2)	7 (2.1)		24.2 (1.5)	47.0 (3.0)	
Readmission within 30 days, *n* (%)											
No	1503 (95.7)	1067 (95.2)	436 (96.9)	0.172^a^	639 (96.5)	317 (95.8)	322 (97.3)	0.396	1501.2 (95.6)	1519.8 (96.6)	0.517
Yes	68 (4.3)	54 (4.8)	14 (3.1)		23 (3.5)	14 (4.2)	9 (2.7)		68.6 (4.4)	54.2 (3.4)	
Postoperative hospitalization time, days^b^											
	7 (6, 9)	8 (6, 10)	6 (5, 8)	<0.001	7 (5, 8)	7 (6, 9)	6 (5, 8)	< 0.001	8.74 (5.30)	8.42 (6.04)	0.43
Hospitalization expenses, Yuan^b^											
	71076 (61443.5, 82746)	65156 (58546, 73875)	85607.5 (78094.25, 95249.75)	<0.001	75631.5 (65510, 86662.5)	66130 (59263.5, 74732.5)	84372 (76326, 95036)	< 0.001	68153.12 (24818.59)	90917.68 (28622.99)	<0.001
Tumour volume, cm^3^ ^b^											
	10 (5, 19)	10 (4, 18)	10 (5, 22)	0.103	10 (5, 23)	10 (5, 21)	11 (5, 24)	0.385	15.41 (20.43)	16.78 (22.95)	0.34
CRM positive, *n* (%)											
No	1562 (99.4)	1118 (99.7)	444 (98.7)	0.02^a^	657 (99.2)	331 (100)	326 (98.5)	0.062^a^	1566.4 (99.8)	1559.6 (99.1)	0.03
Yes	9 (0.6)	3 (0.3)	6 (1.3)		5 (0.8)	0 (0)	5 (1.5)		3.4 (0.2)	14.4 (0.9)	
DRM positive, *n* (%)											
No	1538 (97.9)	1096 (97.8)	442 (98.2)	0.711	641 (96.8)	316 (95.5)	325 (98.2)	0.076	1529.5 (97.4)	1555.3 (98.8)	0.059
Yes	33 (2.1)	25 (2.2)	8 (1.8)		21 (3.2)	15 (4.5)	6 (1.8)		40.3 (2.6)	18.7 (1.2)	
No. harvested lymph nodes^b^											
	14 (11, 18)	14 (11, 17)	16 (12, 21)	<0.001	15 (12, 20)	14 (11, 20)	16 (12, 21)	0.032	15.28 (6.80)	15.77 (6.99)	0.256

APR, abdominoperineal resection; CAA, coloanal anastomosis; CRM, circumferential resection margin; DRM, distal resection margin; IPTW, inverse probability of treatment weight; ISR, intersphincteric resection; LaTME, laparoscopy-assisted total mesorectal excision; PSM, propensity score matching; RaTME, robotic-assisted total mesorectal excision TaTME, transanal total mesorectal excision.

a Fisher exact test.

b Mean (standard deviation) for normal distribution and Median (Q1,Q3) for non-normal distribution.

**Table 3:** Comparison of primary outcome before and after adjustment in different cohorts.

			Crude		Adjusted^a^	
	Total	Events (%)	OR (95% CI)	*P* value	OR (95% CI)	*P*
Entire cohort						
LaTME	1121	59 (5.3)	Ref		Ref	
RaTME	450	38 (8.4)	1.66 (1.09–2.53）	0.019	4.76 (2.46–9.21)	<0.001
PSM cohort						
LaTME	331	15 (4.5)	Ref		Ref	
RaTME	331	31 (9.4)	2.18 (1.15–4.11)	0.017	3.69 (1.63–8.35)	0.002
IPTW cohort						
LaTME	1569.8	76.6 (4.9)	Ref		Ref	
RaTME	1574	145.3 (9.2)	1.98 (1.49–2.64)	<0.001	4.25 (2.91–6.23)	<0.001

ASA, American Society of Anesthesiologists; IPTW, inverse probability of treatment weight; LaTME, laparoscopy-assisted total mesorectal excision; OR, odds ratio; PSM, propensity score matching RaTME, robotic-assisted total mesorectal excision

a Medical centre, sex, age, ASA, application of concurrent radiochemotherapy, distance from the anal verge to the tumour, BMI, conversion to TaTME, pathological stage T, and pathological stage N were adjusted.

The independent factors associated with ISR are shown in Table S2. Overall, a higher incidence of ISR was significantly associated with shorter distance from the anal verge to the tumour [OR, 13.65 (95% CI, 5.98–31.17), *P*<0.001], conversion to TaTME [OR, 26.19 (95% CI, 14.12–48.57), *P*<0.001], and treatment with RaTME [OR, 4.76 (95% CI, 2.46–9.21), *P*<0.001]. Furthermore, female patients were less likely to receive ISR [OR, 0.52 (95% CI, 0.30–0.91), *P*=0.021].

### Secondary outcomes

The secondary outcomes after PSM and IPTW are shown in Table 2. After PSM and IPTW, patients who underwent RaTME had a higher rate of CAA than did those who did not (12.4% versus 6.0% after PSM, *P*=0.007; 11.0% versus 6.2% after IPTW, *P*=0.004). In the RaTME group, anastomosis was more likely to be finished by hand than in the LaTME group (9.7% versus 4.5% after PSM, *P*=0.032; 9.5% versus 4.9% after IPTW, *P*=0.039). Further, RaTME required a longer operative time [172 (146.5, 205] min versus 160 (125, 200.5) min after PSM, *P*<0.001; 187.49±53.49 min versus. 168.86±53.49 min after IPTW, *P*<0.001] and higher hospitalization expenses than did LaTME (*P*<0.001 in both the PSM and IPTW cohorts). The rate of preventive stoma was lower in RaTME after PSM (45.3% versus 56.2% preventive stoma, *P*=0.007) than in LaTME, but this difference did not reach significance after IPTW (*P*=0.711). RaTME was further associated with a shorter postoperative hospitalization time [6 (5,8) versus 7 (6,9), *P*<0.001] and more harvested lymph nodes [16 (12, 21) versus 14 (11, 20), *P*=0.032], but these differences did not retain significance after IPTW (*P*=0.430 and *P*=0.256, respectively). The RaTME group had a higher rate of positive CRM (0.9% versus 0.2%, *P*=0.03) after IPTW, but differences were not significant after PSM (*P*=0.062). No significant difference was found in overall (23.0% versus 19.0% after PSM, *P*=0.252; 27.0% versus 23.3% after IPTW, *P*=0.232) or major complications (26.3% versus 30.2% after PSM, *P*=0.755; 29.1% versus 28.0% after IPTW, *P*=0.859) between RaTME and LaTME. The detailed types of complications are listed in Table S3

### Subgroup analysis

**Figure 1:**
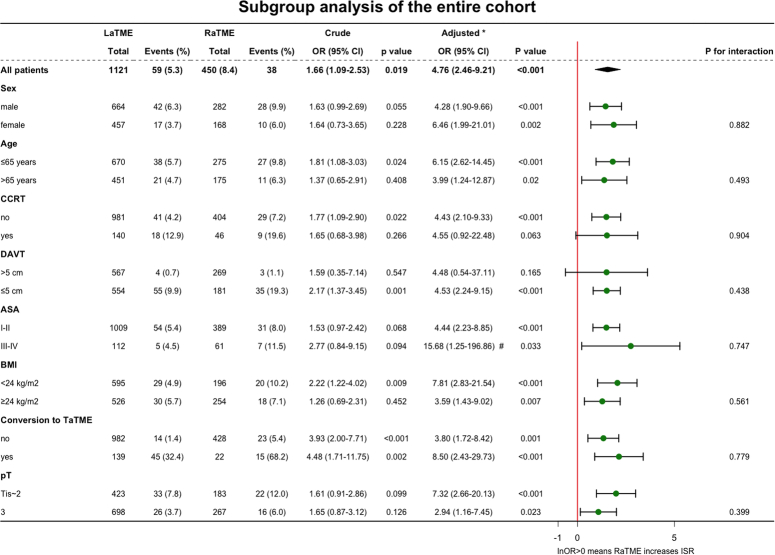
Subgroup analysis of the entire cohort. *Medical centre, sex, age, ASA, application of CCRT, DAVT, BMI, conversion to TaTME, pathological stage T, and pathological stage N were adjusted for. #Medical centre, sex, age, ASA, application of CCRT, BMI, conversion to TaTME, pathological stage T, and pathological stage N were adjusted for. DAVT was not adjusted for in the subgroup of ASA III-IV because no patient with DAVT over 5 cm received ISR in the subgroup of ASA III–IV. ASA, American Society of Anesthesiologists; CCRT, concurrent chemoradiotherapy; DAVT, distance from the anal verge to the tumour; RaTME, robotic-assisted total mesorectal excision; TaTME, transanal total mesorectal excision.

In the subgroup analysis, RaTME was associated with a higher rate of ISR than that of LaTME in the entire population, with an OR of 1.66 (95% CI, 1.09–2.53, *P*=0.019) before adjustment and an OR of 4.76 (95% CI, 2.46–9.21, *P*<0.001) after adjustment. The results were stable in the subgroups based on sex, age, ASA, BMI, conversion to TaTME, and pathological stage T after adjustment. RaTME was associated with a higher rate of ISR when the distance from the anal verge to the tumour was within 5 cm [OR, 2.17 (95% CI, 1.37–3.45), *P*=0.001 before adjustment; OR, 4.53 (95% CI, 2.24–9.15), *P*<0.001 after adjustment]. RaTME may also have the advantage of increasing the ISR rate in patients who did not undergo concurrent chemoradiotherapy before surgery (Fig. 1).

## Discussion

Our study indicates that RaTME is associated with a higher rate of ISR and CAA, and may also require a longer operating time and incur greater hospitalization expenses. However, no significant differences in pathological results or overall complications were observed.

The safety and efficacy of RaTME have been thoroughly investigated in recent years. A lower rate of conversion to open surgery has been reported in both randomized clinical trials (RCT) and meta-analyses^4,16–18^. However, these studies seldom discussed the advantages of RaTME in terms of ultra-low anterior resection and sphincter preservation. ISR is an anus-preserving technique developed as an oncologically safe alternative to APR for low rectal cancers^19,20^. The procedure is conducted by extending the dissection plane of TME through and into the intersphincteric plane. Nonetheless, patients with mid and low rectal cancer may face challenges in resecting the rectum through the abdominal approach or in achieving a clear surgical plane. This is often due to factors such as pelvic narrowing or a tumour obstructing the pelvic inlet, which complicates the surgeon’s ability to dissect to the hiatus of the levator ani muscle and into the intersphincteric plane. Consequently, this may lead to difficulties in CAA and ISR. In extreme cases, some surgeons may need to resort to APR due to challenges in ultra-low anterior resection. Our study revealed that a higher ISR rate is independently associated with RaTME. Furthermore, RaTME is significantly correlated with a higher CAA rate. These findings suggest a preference for RaTME over LaTME in ultra-low anterior resection. In the COLRAR study, a higher ISR rate was observed, with robotic-assisted TME showing 22.5% compared to 16.0% in laparoscopy-assisted TME^5^. However, it is important to note that due to the premature termination of COLRAR owing to poor data accrual, this difference failed to achieve statistical significance. Our results of ISR are consistent with those observed in the REAL study, where a higher ISR rate was also noted in the robotic surgery group^16^. However, in the REAL study, partial ISR was defined as the distal resection line of the internal anal sphincter that did not exceed the dentate line and was performed transabdominally. This definition is confusing and may have resulted in the inclusion of a large proportion of patients with CAA instead of partial ISR. According to the consensus statement from the International Standardization and Optimization Group for intersphincteric resection (ISOG-ISR), partial ISR is defined as a resection at the dentate line^21^. In our study, we clearly distinguished between ISR and CAA to avoid confusion, and we found that RaTME has advantages in both CAA and ISR. Subgroup analysis showed that the advantages of RaTME in ISR existed for all ages and sexes. Even in the subgroup of patients undergoing TaTME, RaTME was still an effective technique for achieving ISR. Multivariate analysis revealed that RaTME and TaTME may be independent factors for increasing ISR.

In this study, three of the four medical centres conducted TaTME surgery when TME could not be completed via the transabdominal approach. TaTME is considered an effective method for achieving TME in patients with a difficult pelvis. In some cases, TaTME may reduce the rate of conversion to laparotomy and increase the rate of anal preservation^22^. In the present study, TaTME was identified as an independent factor that significantly increased the ISR rate. Further, LaTME was associated with a significantly higher rate of conversion to TaTME compared with RaTME in the unmatched population (12.4% versus 4.9%). However, this difference was not significant after PSM (9.7% versus 6.3%) or IPTW (10.8% versus 7.0%). This may be because one of the four medical centres did not implement the TaTME technique. Considering the technical difficulty and long learning curve of TaTME, RaTME may be a promising alternative choice for patients with lower rectal cancer who wish to preserve the anus. In addition to TaTME, RaTME was independently associated with a higher incidence of ISR than that of LaTME in the multivariate analysis. In addition, in the subgroup of patients who underwent TaTME, RaTME was associated with a higher rate of ISR than that of LaTME after adjustment. This may be because RaTME facilitates dissection of the mesorectum above the levator ani muscle, which is more difficult in LaTME. Hence, a higher proportion of LaTME may be converted to TaTME for ultralow anterior resection but not for ISR. This may be the reason why RaTME was still associated with higher ISR (68.2% versus 32.4%) in the TaTME population.

In previous RCT trials, RaTME was thought to be associated with a lower rate of conversion to an open approach^4,16^. This difference was found to be significant in the unmatched cohort in our study but was not significant in the PSM and IPTW cohorts. In our study, RaTME harvested more lymph nodes than LaTME in the unmatched and PSM cohorts. However, according to a prior meta-analysis, the difference in the number of harvested lymph nodes was not significant between the two techniques^18,23^. Similar to other studies, our study found that RaTME and LaTME were comparable in 30-day postoperative complications and major complications with Clavien–Dindo classification III-IV^5,16,24^. Interestingly, the RaTME seemed to be associated with a higher CRM positivity rate in our study, which contradicts the findings of other studies. In most published retrospective and prospective studies, RaTME may have had a lower CRM positivity rate. In our study, a positive CRM was defined as the presence of tumour cells within 1 mm of the CRM on microscopy^25^. Considering the low incidence of positive CRM in our study and the uneven quality control that may exist among pathologists at different medical centres, these results should be interpreted carefully.

This study has several limitations that should be addressed. Previous studies have indicated that RaTME resulted in similar levels of bladder dysfunction, sexual dysfunction, bowel recovery, and quality of life compared with LaTME^4,5,24^. However, we did not investigate these outcomes in the present study. In addition, our study did not record ISR subtypes because the data were collected retrospectively. Moreover, as this was a retrospective analysis, inherent selection bias and group imbalance could not be avoided. Although PSM and IPTW were conducted to ensure an extent of compatibility of groups, and multivariate analysis was also performed in the analysis of primary outcomes, pre-inclusion selection bias likely occurred and may have resulted in the exclusion of patients with an unfavourable risk profile. Additionally, unknown risk factors or factors not included in the propensity score model and multivariate analysis may have been unbalanced. Furthermore, when surgeons communicated with patients about RaTME and LaTME prior to the patients making a decision, surgeons might have emphasized the advantages and disadvantages of RaTME. Specifically, they may have highlighted its superior three-dimensional vision and flexible robotic arms, which can facilitate precise operations, while also mentioning the higher hospitalization expenses associated with RaTME. These higher expenses of RaTME could play a significant role in the patients’ decision-making process, potentially leading to bias in this study. Finally, data regarding the survival benefit was not available in our study because long-term follow-up was incomplete. Thus, although our study indicates that RaTME may be a good choice for middle and low rectal cancer with the advantage of increasing the ISR rate, it cannot yet be considered a gold standard because of the lack of information regarding survival benefits and functional results.

## Conclusion

In conclusion, our study showed that RaTME may facilitate ISR in middle and low rectal cancers, with pathological outcomes and postoperative complications comparable to those of LaTME. Thus, we suggest that RaTME may be an alternative method to achieve ISR in addition to TaTME. However, it should be considered that RaTME may also involve longer operating times and higher hospitalization expenses, and the long-term survival benefits require further investigation.

## Ethical approval

This study was approved by the Ethics Committee of the First Hospital of Jilin University (approval numer: 23K163-001). The detailed procedures were explained to all patients, and written informed consent was obtained prior to treatment.

## Source of funding

None.

## Author contribution

Y.G. is the first author. He designed the study, drafted the article, and made statistical analysis. L.H. collected the data, analyzed data and interpreted data. W.T., S.R., Z.C. revised the article and interpreted data. K.T., B.W., C.L. help collect the data, interpret the data and created digital artwork. W.T., S.R., Z.C. and Q.W. contributed cases. Q.W. is the corresponding authors. He designed the study, interpreted data and revised the article. Final approval of manuscript is done by all authors.

## Conflicts of interest disclosure

The authors declare that they have no competing interests.

## Research registration unique identifying number (UIN)

1. Name of the registry: ResearchRegistry.com
2. Unique Identifying number or registration ID: researchregistry9626
3. Hyperlink to your specific registration (must be publicly accessible and will be checked): https://researchregistry.knack.com/researchregistry#home/registrationdetails/6532087a864dfa00295a6973/.

## Guarantor

Yuchen Guo and Quan Wang are the guarantors.

## Data statement

The research data can be available on the request of reviewers and other investigators by email.

## Supplementary Material

SUPPLEMENTARY MATERIAL
