# Supplementary material for: Intersphincteric resection following robotic-assisted versus laparoscopy-assisted total mesorectal excision for middle and low rectal cancer: a multicentre propensity score analysis of 1571 patients
Source: Int J Surg. 2024 Jan 19;110(4):1904–12. doi: 10.1097/JS9.0000000000001053 (PMC11020017; doi:10.1097/JS9.0000000000001053)
Supplement: SUPPLEMENTARY MATERIAL [file js9-110-1904-s001.docx]

**Supplementary Materials - Index**

**Supplementary Methods**

Definitions *pag.2-3*

**Supplementary Figures and Tables**

Figure S1 Flow diagram of enrollment *pag.4*

Table S1 Population and outcomes of each medical centers after PSM *pag.5*

Table S2 Analysis of the risk factors of ISR in entire cohort *pag.6*

Table S3 Types of complications in detail *pag.7*

**References** *pag.8*

**Supplementary Methods**

**Definitions**

The distance from the anal verge to the tumor was evaluated using preoperative rectal nuclear magnetic resonance imaging (NMRI), endorectal ultrasonography, colonoscopy, or digital rectal examination. When the values measured using the different methods differed, the minimum value was used. Regarding pathological outcomes, CRM positivity was defined as the presence of tumor cells within 1 mm of the CRM on microscopy (1). DRM positivity was defined as a distal margin within 1 mm of the tumor. Tumor volume was evaluated according to the pathological report, and calculated by multiplying the length, width, and thickness of the tumor following formalin fixation. The pathological TNM classification was determined according to the American Joint Committee on Cancer (AJCC) Cancer Staging Manual, 8^th^ edition. The operating time was defined as the time from cutting the skin to suturing the skin or performing an enterostomy. Intraoperative hemorrhage was defined as intraoperative bleeding of > 200 ml.

Postoperative complications included fever of unknown origin, bowel obstruction, anastomotic leakage, surgical site infection (SSI), other incisional complications, respiratory complications, urinary complications, cardiovascular and cerebrovascular complications, diarrhea, chylous fistula, intraperitoneal hemorrhage, digestive hemorrhage, gastroparesis, and others (including bacteremia, cholecystitis, ion discharge, pancreatitis, and mental and behavioral abnormalities). Complications were graded according to the Clavien–Dindo classification (2). The diagnosis of anastomotic leakage was based on the patient’s postoperative clinical symptoms, signs of abdominal infection, or drainage properties, and was confirmed by imaging (angiography or computed tomography) or secondary surgery. Postoperative intraperitoneal/digestive hemorrhage was defined as bloody drainage/bloody stool of > 100 mL in 1 h, or evidence of hemorrhage on CT/endoscopy/angiography. The respiratory complications included pulmonary or pleural effusion. Urinary complications included infection, retention, and leakage. SSI was defined according to the U.S. Centers for Disease Control and Prevention guidelines (3,4). Other incisional complications included bleeding, hernia and disruption.

.

**Supplemental Figures and Tables**


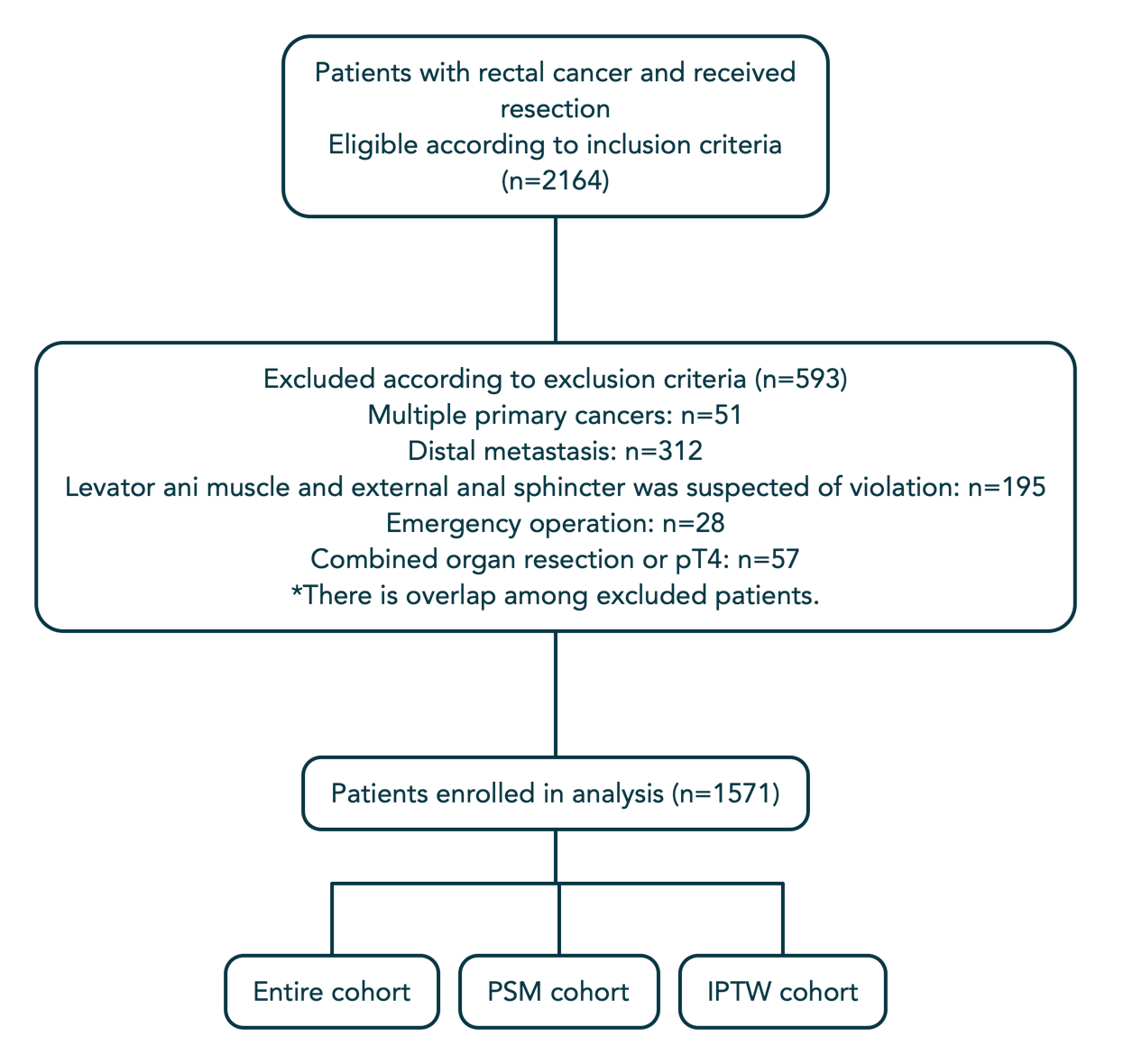


Figure S1 Flow diagram of enrollment

**References**

1. Nagtegaal ID, Quirke P. What is the role for the circumferential margin in the modern treatment of rectal cancer? J Clin Oncol. 2008 Jan 10;26(2):303–12.

2. Dindo D, Demartines N, Clavien PA. Classification of Surgical Complications. Ann Surg. 2004 Aug;240(2):205–13.

3. Mangram AJ, Horan TC, Pearson ML, Silver LC, Jarvis WR. Guideline for Prevention of Surgical Site Infection, 1999. Centers for Disease Control and Prevention (CDC) Hospital Infection Control Practices Advisory Committee. Am J Infect Control. 1999 Apr;27(2):97–132; quiz 133–4; discussion 96.

4. Berríos-Torres SI, Umscheid CA, Bratzler DW, Leas B, Stone EC, Kelz RR, et al. Centers for Disease Control and Prevention Guideline for the Prevention of Surgical Site Infection, 2017. JAMA Surg. 2017 Aug 1;152(8):784–91.
